# Kernel principal component analysis (PCA) control chart for monitoring mixed non-linear variable and attribute quality characteristics

**DOI:** 10.1016/j.heliyon.2022.e09590

**Published:** 2022-06-06

**Authors:** Muhammad Ahsan, Muhammad Mashuri, Hidayatul Khusna, Wibawati

**Affiliations:** Department of Statistics, Institut Teknologi Sepuluh Nopember, Surabaya, Indonesia

**Keywords:** Kernel PCA, $T^{2}$ Hotelling's chart, Mixed quality characteristics, Kernel Density Estimation, Nonlinearity

## Abstract

The products are commonly measured by two types of quality characteristics. The variable characteristics measure the numerical scale. Meanwhile, the attribute characteristics measure the categorical data. Furthermore, in monitoring processes, the multivariate variable quality characteristics may have a nonlinear relationship. In this paper, the Kernel PCA control chart is applied to monitor the mixed (attribute and variable) characteristics with the nonlinear relationship. First, the Average Run Length (ARL) is utilized to evaluate the performance of the proposed chart. The simulation studies show that the proposed chart can detect the shift in process. For this case, the Radial Basis Function (RBF) kernel demonstrates the consistent performance for several cases studied. Second, the performance comparison between the proposed chart and the conventional PCA Mix chart is performed. Based on the results, it is known that the proposed chart performs better in detecting the small shift in process. Finally, the proposed chart is applied to monitor the well-known NSL KDD dataset. The proposed chart shows good accuracy in detecting intrusion in the network. However, it still produces more False Negatives (FN).

## 1 Introduction

Two types of control charts have been developed based on the monitored quality characteristics. These charts are named as the attribute and variable charts. The variable control chart is developed to monitor the variable quality characteristics (in variable or ratio scale) such as length, temperature, or height (Montgomery, 2009). Meanwhile, to monitor the attribute quality characteristics (in categorical scale) the attribute chart was applied (Ahsan et al., 2018). When the characteristics quality is correlated or cannot be monitored separately, the multivariate control chat has been developed. There are three main types of multivariate variable control charts namely Shewhart, multivariate exponentially weighted moving average (MEWMA), and multivariate cumulative sum (MCUSUM).

The product quality characteristics are not only gauged individually by the attribute or variable characteristics but also can be monitored using a mixed scheme. In order to facilitate a mixed procedure of the monitoring process, several works have studied the development of the mixed characteristics charts. The mixed scheme by employing the combination between $\bar{X}$ and *np* charts has been proposed and has a good performance in monitoring mixed characteristics (Aslam et al., 2015). The mixed chart proposed by Aslam et al. (2015) is compared with Hybrid Exponential Weighted Moving Average (HEWMA) (Aslam et al., 2016). The spatial-sign covariance matrix-based control chart has been proposed by integrating the standardized ranks and spatial signs in calculating the mixed statistics (Wang et al., 2018). Furthermore, the principal component analysis for mixed data is applied in inspecting the process (Ahsan et al., 2018) and in detecting outliers (Ahsan et al., 2019). To overcome the PCA Mix chart drawbacks, Ahsan et al. (2020) proposed the Kernel PCA (KPCA) Mix chart for monitoring the mixed variable and attribute quality characteristics.

The problem arises when the PCA Mix chart (Ahsan et al., 2018) is applied to inspect the nonlinear multivariate processes. In monitoring processes, the multivariate quality characteristics may have a nonlinear relationship. Some studies about the utilization of control charts in detecting a shift in nonlinear data have been conducted. A multivariate chart based on KPCA and Exponentially Weighted Moving Average (EWMA) is proposed to monitor nonlinear biological processes (Yoo and Lee, 2006). Khediri et al. (2010) suggested Support Vector Regression (SVR) control charts for multivariate nonlinear processes with dependency on its samples. Fan et al. (2014) proposed a control chart based on filtering kernel independent component analysis–principal component analysis (FKICA–PCA) to monitor multivariate industrial processes. The nonparametric Revised Spatial Rank Exponential Weighted Moving Average (RSREWMA) control chart is developed to assess the multivariate nonlinear profile data (Pan et al., 2019). Kernel PCA can be applied in monitoring such cases mentioned above by using the control chart approach.

Based on the previous study, the KPCA Mix chart (Ahsan et al., 2020) can be extended to monitor the multivariate nonlinear data. Therefore, this research suggests a mixed multivariate control chart based on the KPCA algorithm that can accommodate the mixed type of quality characteristics with the nonlinear relationship. The estimated PCs Mix from KPCA are then transformed into Hotelling's $T^{2}$ statistics. The control limit of $T^{2}$ statistics is calculated using the kernel density estimation (KDE), the same method used in Ahsan et al. (2020). Moreover, to show the benefits and drawbacks of the proposed chart, its performance is compared with the conventional PCA Mix chart. The rest of this article is arranged as follows: Some related studies are shown in section 2. Section 3 describes the Kernel PCA method. The charting procedures of the proposed KPCA Mix control chart are displayed in section 4. Section 5 presents the performance assessment of the proposed chart in detecting a shift in the process along with the comparison with the PCA Mix chart. The utilization of the proposed chart in simulated and real data is shown in Section 6. Some conclusions and possible future research are presented in Section 7.

## 2 Related research

The recent studies of the control charts are presented in this section. There are three main categories of control charts discussed in this section such as a multivariate variable chart, attribute chart, and mixed chart. The recent developments in multivariate variable charts are displayed in Table 1. Table 2 shows the recent developments of multivariate attribute charts. Meanwhile, the recent developments in mixed characteristics are presented in Table 3.

**Table 1:** The recent development of multivariate variable control charts.

Sources	Proposed scheme	Findings
Chiang et al. (2021)	New scheme of multivariate auxiliary-information-based (AIB) chart	The performance of the proposed chart is evaluated using Monte-Carlo simulation and applied to cement data
Ahmad and Ahmed (2021)	*T*^2^ control chart to inspect the high dimensional data	The proposed method is usable without preprocessing or dimension reduction with high accuracy detection
Haddad (2021)	*T*^2^ control charts using modified Mahalanobis distance	The proposed method has better performance in detecting more outliers compared to the traditional chart
Cabana and Lillo (2021)	Robust multivariate chart for individual observations using reweighted shrinkage estimators	The proposed chart has a better performance for high dimensional and high contaminated data
Maleki et al. (2020)	Median estimators of the *T*^2^ control chart	The proposed method outperforms performance compared to the conventional chart
Haddad et al. (2019)	Bivariate Hotelling's *T*^2^ charts with bootstrap data	The proposed method shows a better performance compared to the conventional method
Tiengket et al. (2020)	Bivariate Copulas on the Hotelling's *T*^2^ Control Chart	The bivariate copulas method can be used in the Hotelling's *T*^2^ chart
Mashuri et al. (2019)	*Tr* (*R*^2^) control charts with Kernel Density Estimation (KDE) control limit	The proposed control chart method presents better performance to detect the shift for the large characteristics and sample size
Mehmood et al. (2019)	Hotelling *T*^2^ control chart based on bivariate ranked set schemes	Proposed control chart schemes demonstrate an outstanding performance compared to the classical Hotelling *T*^2^
Haq and Khoo (2019)	Adaptive MEWMA chart	The proposed chart surpasses the performances of the existing adaptive multivariate charts
Flury and Quaglino (2018)	MEWMA chart for asymmetric gamma distributions	The proposed MEWMA chart outperforms the performance of the conventional *T*^2^ chart in all the cases
Haq et al. (2020)	Dual MCUSUM charts with auxiliary information for the process mean	The proposed chart has a better performance compared to the DMCUSUM and MDMCUSUM charts when detecting different sizes of a shift in the process mean vector

**Table 2:** The recent development of attribute control charts.

Sources	Proposed scheme	Findings
Yeganeh et al. (2021)	Combined novel run rules and MEWMA control chart	The proposed method has better performance for small and moderate shifts in monitoring linear profiles
Xie et al. (2021)	MCUSUM control chart for monitoring Gumbel's bivariate exponential data	The proposed chart outperforms the other charts for most shift domains
Mashuri et al. (2020)	Fuzzy bivariate chart	The proposed chart is more sensitive than the conventional bivariate Poisson chart
Zhou et al. (2020)	Synthetic control chart for attribute inspection	The proposed chart demonstrates a higher detection performance for small and large mean shifts
Quinino et al. (2020)	Attribute chart for the joint monitoring of mean and variance	The proposed method is easier to be implemented compared to the conventional approach
Aldosari et al. (2019)	Attribute control chart for multivariate Poisson distribution using multiple dependent state repetitive sampling (MDSRS)	The proposed method has a better performance than the conventional one based on repetitive sampling
Aslam et al. (2019)	Shewhart attribute control with the neutrosophic statistical interval	The proposed attribute control chart has a good ability to detect a shift in the process
Chong et al. (2019)	Multi-attribute CUSUM-np chart	The proposed procedure has a better or equal performance compared to the conventional chart
Aslam (2019)	Attribute control chart using the repetitive sampling under the fuzzy neutrosophic system	The proposed chart with repetitive sampling under the fuzzy neutrosophic system is more sensitive in detecting a shift in the process as compared with the existing chart
Lee et al. (2017)	Multinomial generalized likelihood ratio (MGLR) chart	The proposed chart has better performance than the set of 2-sided Bernoulli CUSUM charts

**Table 3:** The recent development in the mixed variable and attribute control charts.

Sources	Proposed scheme	Findings
Ahsan et al. (2020)	Kernel PCA Mix Chart	The proposed chart has a better performance compared to the PCA Mix chart
Ahsan et al. (2019)	PCA Mix chart for detecting outlier in mixed characteristics scheme	The proposed chart has a great performance to detect more outliers with a higher percentage of outliers added compared to the conventional and other robust charts
Ahsan et al. (2018)	PCA Mix control chart	The proposed chart presents good performance for an appropriate number of principal components used
Wang et al. (2018)	Multivariate sign chart	Simulations show the superiority of the proposed control chart in monitoring mixed-type data
Aslam et al. (2015)	The mixed chart to monitor the process	The mixed chart shows excellent performance in the monitoring process

Based on the recent development of the mixed control chart, it can be seen that there are a few works that studied the mixed monitoring variable and attribute characteristics. Therefore, more development in this area is needed especially for nonlinear data. This work proposes the mixed control chart based on the Kernel PCA Mix algorithm. The control limit of the $T^{2}$ statistics from PCs Mix is estimated using the KDE method which has better performance in estimating the non-normal data. The proposed chart is expected to have better performance to monitor the nonlinear mixed data. To show this, the performance of the proposed chart is compared with the conventional PCA Mix chart. Also, the application to the real data is conducted.

## 3 Kernel PCA

PCA is the basis of transformation to diagonalize the estimated covariance matrix **C** from input data. PCA was originally proposed for linear data. Therefore, this method is not powerful for nonlinear data. To overcome this nonlinearity problem, Schölkopf et al. (1997) proposed the Kernel PCA scheme.

**Figure 1:**
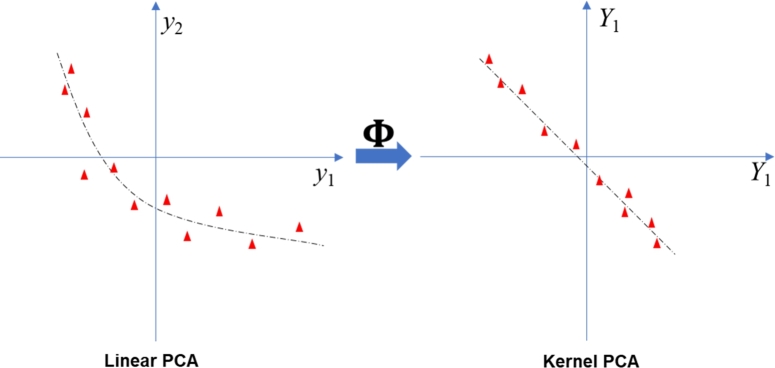
Illustration of KPCA.

The basic idea of Kernel PCA is calculating the Principal Component Scores in higher dimensional space by conducting a nonlinear mapping $\Phi :R^{p}\to F,y\mapsto Y$ as displayed in Fig. 1. This mapping can be executed by utilizing the kernel functions known from the Support Vector Method (SVM) (Boser et al., 1992).

Assume that the centered data are mapped to feature space *F*, $\Phi (y_{1}),...,\Phi (y_{n})$. The feature space covariance matrix with a size of n×n can be written as in Equation (3.1).

(3.1) $$C^{F}=\frac{1}{n}\sum_{j=1}^{n}\Phi (y_{j})\Phi {\left(y_{j}\right)}^{T}.$$

The next step is estimating the eigenvalues λ≥0 eigenvector that satisfies Equation (3.2).

(3.2) $$\lambda V=C^{F}V.$$

In general, the mapping Φ(.) is not always can be calculated. To solve the problem, the dot product calculation from to vector in feature space is performed. Let **K** with a size of n×n defined as $K_{ij}=\langle \Phi (y_{i}),\Phi (y_{j})\rangle$. The Principal component score (PCs) **t** is computed using projection of $\Phi (y_{i})$ to eigenvector $V_{v}$, where v=1,2,...,l, as expressed in Equation (3.3).

(3.3) $$t_{v}=\langle V_{v},\Phi (y)\rangle =\sum_{i=1}^{n}\alpha_{i}^{v}\langle \Phi (y_{i}),\Phi (y)\rangle .$$

To solve the eigenvalue problem and principal component calculation, nonlinear mapping is not needed to be conducted. To replace this, the kernel function can be constructed $K\left(y_{i},y\right)=\langle \Phi (y_{i}),\Phi (y)\rangle$.

## 4 Kernel PCA Mix chart

### 4.1 Statistics calculation

The main concept of the Kernel PCA Mix chart is to form the **Z** as a representation of the mixed variable. There are two main steps in the KPCA Mix chart procedure. First, the $T^{2}$ statistics are computed from matrix **Z**. Second, the control limit calculation is performed by applying the KDE. These procedures are illustrated by the flowchart in Fig. 1. Furthermore, detailed procedures are given as follows:

**Statistics** $T^{2}$ **calculation**

1. Create matrix $Z=[Z_{1},Z_{2}]$ sized n×(p+m) where:
  a. $Z_{1}$ is the centered version of a matrix $Y_{1}$ which is contained the variable characteristics (numeric data).
  b. $Z_{2}$ is the centered version of a matrix **B** which is contained the dummy from each category in attribute characteristics (categorical data) $Y_{2}$.
2. Define $N=\frac{1}{n}I_{n}$, where $I_{n}$ is the identity matrix with the size of n×n.
3. Define $M=\mathrm{diag}\left(1,...,1,\frac{n}{n_{1}},...,\frac{n}{n_{m}}\right)$, where the first *p* columns are specified as by 1 and the last *m* columns are weighted by $\frac{n}{n_{s}}$, for s=1,2,…,m.
4. Calculate $\overset{˜}{Z}=N^{\frac{1}{2}}{\mathrm{ZM}}^{\frac{1}{2}}$.
5. Calculate the matrix kernel $K=K({\overset{˜}{z}}_{i},{\overset{˜}{z}}_{j})=\langle \Phi ({\overset{˜}{z}}_{i}),\Phi ({\overset{˜}{z}}_{j})\rangle$.
6. Calculate Principal Component Scores (PCs) **t** using the formula as shown in Equation (4.4).
  (4.4) $$t_{v}=\sum_{i=1}^{n}{\overset{˜}{\alpha }}_{i,v}\langle \Phi (z_{i}),\Phi (z)\rangle =\sum_{i=1}^{n}{\overset{˜}{\alpha }}_{i,v}\overset{˜}{K}(z_{i},z).$$
7. From the first *l* principal component **t**, calculate the $T^{2}$ statistics using Equation (4.5).
  (4.5) $${\overset{˜}{T}}_{k}^{2}=\sum_{v=1}^{l}t_{v}\lambda_{v}^{-1}t_{v}^{T},$$
  where v=1,2,...,l, and $\lambda_{v}$ eigenvalues that correspond to *v*-th PCs.

### 4.2 Control limit calculation

The control limit is estimated using the KDE approach due to its ability to follow the unknown distribution of data input. The procedures of control limit calculation are presented as:

1. Estimate the empirical density of ${\overset{˜}{T}}_{k}^{2}$ statistics using Equation (4.6).
  (4.6) $${\overset{ˆ}{f}}_{h}({\overset{˜}{T}}_{k}^{2})=\frac{1}{n\overset{ˆ}{h}}\sum_{i=1}^{n}k\left(\frac{T^{2}-{\overset{˜}{T}}_{k,i}^{2}}{\overset{ˆ}{h}}\right).$$
2. Calculate ${\overset{ˆ}{F}}_{h}({\overset{˜}{t}}_{k})=\int_{0}^{{\overset{˜}{t}}_{k}}{\overset{ˆ}{f}}_{h}({\overset{˜}{T}}_{k}^{2})d{\overset{˜}{T}}_{k}^{2}$ using the numerical integration trapezoid rule as in Equation (4.7).
  (4.7) $$\int_{\pi_{\min}}^{\pi_{\max}}{\overset{ˆ}{f}}_{h}({\overset{˜}{T}}_{k}^{2})d{\overset{˜}{T}}^{2}\approx \frac{\pi_{\max}-\pi_{\min}}{2n}\sum_{i=1}^{n}\left({\overset{ˆ}{f}}_{h}({\overset{˜}{T}}_{k,i}^{2})+{\overset{ˆ}{f}}_{h}({\overset{˜}{T}}_{k,i+1}^{2})\right),$$
  where $\pi_{\min}$ and $\pi_{\max}$ are the maximum and minimum values of ${\overset{˜}{T}}_{k}^{2}$.
3. Calculate the control limit using the expression as shown in Equation (4.8).
  (4.8) $$\overset{˜}{\mathrm{CL}}={\overset{ˆ}{F}}_{h}^{-1}({\overset{˜}{t}}_{k})(1-\alpha ).$$

## 5 Performance evaluation

### 5.1 Simulation set-up

The performance of the proposed control chart is assessed for the variable characteristics (numeric data) which have a nonlinear relationship. The nonlinear data is generated using the following procedures:

1. Generate vector $y_{0}∼N(0,1)$ and $a_{0}∼U(0,01,1)$.
2. Define five nonlinear variable characteristics as:
  $$y_{1}^{nl}=a_{0}+y_{0}\;y_{2}^{nl}=2x_{1}^{nl}-{\left(y_{1}^{nl}\right)}^{2}+4{\left(y_{1}^{nl}\right)}^{3}+y_{0}\;y_{3}^{nl}=\exp(y_{1}^{nl})+y_{0}\;y_{4}^{nl}=\sin(y_{1}^{nl})-3\sin\left({\left(y_{1}^{nl}\right)}^{4}\right)+y_{0}\;y_{5}^{nl}=2{\left(y_{1}^{nl}\right)}^{2}-2\cos\left({\left(y_{1}^{nl}\right)}^{2}\right)+y_{0}.$$

**Figure 2:**
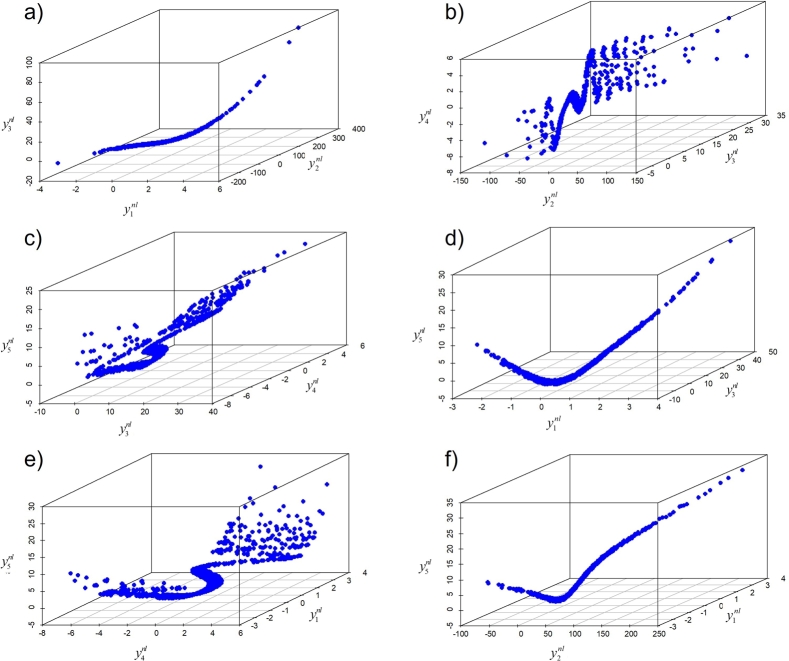
3D Scatter plot of generated nonlinear data: a) $y_{1}^{nl},y_{2}^{nl}$, and, $y_{3}^{nl}$, b) $y_{2}^{nl},y_{3}^{nl}$, and, $y_{4}^{nl}$, c) $y_{3}^{nl},y_{4}^{nl}$, and, $y_{5}^{nl}$, d) $y_{1}^{nl},y_{3}^{nl}$, and, $y_{5}^{nl}$, e) $y_{1}^{nl},y_{4}^{nl}$, and, $y_{5}^{nl}$, f) $y_{1}^{nl},y_{2}^{nl}$, and, $y_{5}^{nl}$.

The visualizations of those five generated characteristics are presented in Fig. 2.

### 5.2 Performance evaluation

The number of variable quality characteristics $Y_{1}$ (generated from the Multivariate Normal distribution) involved is five. Meanwhile, the number of principal components *l* evaluated is 2, 3, and 4. The performance is evaluated for three cases, namely the case of attribute characteristics $Y_{2}$ (generated from the Multinomial distribution) with extreme imbalanced, imbalanced, and balanced proportions as defined below:

a. Balanced case with parameter $\theta_{1},\theta_{2}=0.3\text{ and }\theta_{3}=0.4$
b. Imbalanced case with parameter $\theta_{1},\theta_{2}=0.1\text{ and }\theta_{3}=0.8$
c. Extreme Imbalanced case with parameter $\theta_{1},\theta_{2}=0.05\text{ and }\theta_{3}=0.9$

Furthermore, three categories of kernel functions utilized in this research are defined as follows:

a. Linear: $K(x_{i},x_{j})=\langle x_{i},x_{j}\rangle$.
b. Polynomial: $K(x,y)={\left(\langle x,y\rangle +1\right)}^{d}$.
c. Radial Basis Function (RBF): $K(x_{i},x_{j})=\exp\left(-\sigma^{⁎}{\left\|x_{i}-x_{j}\right\|}^{2}\right)$.

#### 5.2.1 Extreme imbalanced case

The performance of the Kernel PCA Mix chart in handling nonlinear data with an extreme imbalanced proportion of attribute characteristics is tabulated in Table 4, Table 5, Table 6. For the small number of the principal component score used, it is seen that the RBF kernel performs poorer compared to the other kernels. Meanwhile, for the larger number of the principal component score used, the RBF kernel displays better results compared to the other functions. Also, for this case, the KDE control limit produces stable ARL_0_ at about 370.

**Table 4:** ARLs of an extreme imbalanced case for *l* = 2.

Shift		Kernel functions		
*δ*_*S*_	*δ*_*μ*_	RBF	Polynomial	Linear
0	0	**376.820**	**374.850**	**379.000**
0.1	0.0025	367.375	377.570	375.855
0.2	0.0050	357.063	354.560	368.283
0.3	0.0075	313.003	345.998	365.330
0.4	0.0100	284.322	330.686	346.508
0.5	0.0125	264.272	317.742	327.998
0.6	0.0150	250.244	302.643	310.600
0.7	0.0175	236.421	286.088	293.735
0.8	0.0200	226.051	268.144	274.916
0.9	0.0225	220.402	252.661	261.438
1.0	0.0250	219.707	238.942	246.952
1.1	0.0275	224.183	225.516	233.486
1.2	0.0300	239.949	213.429	221.341
1.3	0.0325	272.919	202.299	209.421
1.4	0.0350	310.705	191.916	199.267
1.5	0.0375	352.232	182.158	189.546

**Table 5:** ARLs of an extreme imbalanced case for *l* = 3.

Shift		Kernel functions		
*δ*_*S*_	*δ*_*μ*_	RBF	Polynomial	Linear
0.1	0.0025	**370.920**	**380.330**	**391.740**
0.2	0.0050	361.410	362.590	380.240
0.3	0.0075	356.220	363.143	387.913
0.4	0.0100	323.920	340.355	382.750
0.5	0.0125	303.690	336.754	365.910
0.6	0.0150	281.498	319.845	341.658
0.7	0.0175	267.280	308.166	323.410
0.8	0.0200	252.489	294.769	306.358
0.9	0.0225	235.111	282.124	287.041
1.0	0.0250	220.115	268.235	269.949
1.1	0.0275	207.927	252.926	256.348
1.2	0.0300	196.856	240.774	242.078
1.3	0.0325	186.622	227.922	228.676
1.4	0.0350	177.566	214.832	216.686
1.5	0.0375	169.523	204.300	205.981

**Table 6:** ARLs of an extreme imbalanced case for *l* = 4.

Shift		Kernel functions		
*δ*_*S*_	*δ*_*μ*_	RBF	Polynomial	Linear
0	0	**362.860**	**376.820**	**388.540**
0.1	0.0025	365.750	405.895	444.445
0.2	0.0050	359.500	410.370	427.713
0.3	0.0075	350.493	406.170	421.805
0.4	0.0100	338.492	397.478	404.936
0.5	0.0125	321.630	381.345	381.178
0.6	0.0150	311.320	358.761	362.487
0.7	0.0175	297.746	341.734	342.331
0.8	0.0200	285.544	320.178	327.656
0.9	0.0225	274.721	305.700	314.535
1.0	0.0250	260.177	293.063	299.053
1.1	0.0275	248.052	280.185	284.094
1.2	0.0300	236.200	266.963	270.278
1.3	0.0325	224.626	254.882	258.745
1.4	0.0350	214.449	243.619	247.118
1.5	0.0375	205.602	233.453	235.817

#### 5.2.2 Imbalanced case

Table 7, Table 8, Table 9 show the Kernel PCA Mix chart performance in inspecting the nonlinear for an extreme imbalanced proportion of attribute characteristics. Similar to the previous results, the control limit produces stable ARL_0_ at about 370. For all number of principal component scores used, the RBF kernel has a preferable performance compared to the other functions. It is also known that the linear kernel displays poorer results in this case.

**Table 7:** ARLs of the imbalanced case for *l* = 2.

Shift		Kernel		
*δ*_*S*_	*δ*_*μ*_	RBF	Polynomial	Linear
0	0	**386.060**	**367.300**	**380.950**
0.1	0.0025	346.665	349.770	384.000
0.2	0.0050	306.600	328.840	379.383
0.3	0.0075	268.633	327.043	366.278
0.4	0.0100	242.388	317.712	348.862
0.5	0.0125	222.198	302.458	333.512
0.6	0.0150	208.613	284.601	314.729
0.7	0.0175	193.365	266.913	295.940
0.8	0.0200	182.924	250.563	277.669
0.9	0.0225	175.184	235.500	262.847
1.0	0.0250	172.916	222.804	246.770
1.1	0.0275	172.819	209.485	233.871
1.2	0.0300	176.240	198.273	220.032
1.3	0.0325	175.111	187.549	207.769
1.4	0.0350	167.725	178.290	197.263
1.5	0.0375	159.685	169.472	187.162

**Table 8:** ARLs of an imbalanced case for *l* = 3.

Shift		Kernel		
*δ*_*S*_	*δ*_*μ*_	RBF	Polynomial	Linear
0	0	**371.020**	**359.550**	**396.730**
0.1	0.0025	369.610	376.185	425.675
0.2	0.0050	355.200	374.097	423.697
0.3	0.0075	353.843	369.205	422.503
0.4	0.0100	331.568	358.198	400.838
0.5	0.0125	306.777	351.158	377.570
0.6	0.0150	284.471	335.774	355.724
0.7	0.0175	264.586	319.088	336.219
0.8	0.0200	248.086	301.681	317.538
0.9	0.0225	233.595	284.584	299.487
1.0	0.0250	220.216	269.831	281.449
1.1	0.0275	207.939	256.110	265.438
1.2	0.0300	197.140	242.057	250.743
1.3	0.0325	187.698	228.902	238.136
1.4	0.0350	178.887	217.107	226.352
1.5	0.0375	161.626	206.726	214.664

**Table 9:** ARLs of an imbalanced case for *l* = 4.

Shift		Kernel		
*δ*_*S*_	*δ*_*μ*_	RBF	Polynomial	Linear
0	0	**371.100**	**394.810**	**377.530**
0.1	0.0025	351.615	382.655	396.125
0.2	0.0050	337.440	360.083	401.523
0.3	0.0075	335.985	345.143	395.915
0.4	0.0100	322.286	329.336	381.536
0.5	0.0125	308.940	309.580	363.160
0.6	0.0150	296.383	295.949	344.946
0.7	0.0175	279.708	278.995	325.604
0.8	0.0200	264.274	265.733	306.423
0.9	0.0225	251.411	252.864	287.762
1.0	0.0250	238.127	239.604	273.223
1.1	0.0275	226.427	228.050	260.837
1.2	0.0300	217.344	218.267	248.189
1.3	0.0325	207.195	207.876	236.569
1.4	0.0350	197.691	198.643	225.320
1.5	0.0375	188.935	189.732	215.198

#### 5.2.3 Balanced case

Kernel PCA Mix chart performance in assessing the nonlinear data with a balanced proportion of attribute characteristics is displayed in Table 10, Table 11, Table 12. Similar to the previous results, the control limit produces consistent ARL_0_ at about 370. The RBF kernel performs better compared to the others for all number of principal component scores used. Also, the RBF kernel reaches its peak performance when inspecting the balanced proportion of attribute characteristics. For this case, the Polynomial and Linear kernel functions have similar performance.

**Table 10:** ARLs of a balanced case for *l* = 2.

Shift		Kernel		
*δ*_*S*_	*δ*_*μ*_	RBF	Polynomial	Linear
0	0	**380.770**	**398.270**	**351.040**
0.1	0.0025	364.740	426.900	363.020
0.2	0.0050	317.727	404.150	370.863
0.3	0.0075	281.193	388.378	358.250
0.4	0.0100	257.002	375.390	346.804
0.5	0.0125	239.968	353.718	335.335
0.6	0.0150	224.706	333.024	312.767
0.7	0.0175	210.456	310.153	293.535
0.8	0.0200	204.304	290.936	276.356
0.9	0.0225	197.367	272.970	259.842
1.0	0.0250	198.296	256.436	245.284
1.1	0.0275	187.847	242.783	231.725
1.2	0.0300	184.638	229.729	218.880
1.3	0.0325	173.244	217.334	206.827
1.4	0.0350	171.971	205.771	196.301
1.5	0.0375	160.653	195.590	186.618

**Table 11:** ARLs of a balanced case for *l* = 3.

Shift		Kernel		
*δ*_*S*_	*δ*_*μ*_	RBF	Polynomial	Linear
0	0	**374.770**	**365.750**	**385.610**
0.1	0.0025	368.130	412.890	389.070
0.2	0.0050	349.987	402.257	384.577
0.3	0.0075	318.833	389.743	379.578
0.4	0.0100	294.130	375.072	359.124
0.5	0.0125	274.145	352.745	340.432
0.6	0.0150	256.280	338.009	320.169
0.7	0.0175	245.261	314.724	301.196
0.8	0.0200	230.261	293.350	284.602
0.9	0.0225	218.263	276.424	270.494
1.0	0.0250	207.781	259.397	257.344
1.1	0.0275	196.715	243.654	241.558
1.2	0.0300	187.601	229.277	227.823
1.3	0.0325	178.948	216.039	215.006
1.4	0.0350	170.626	204.779	204.089
1.5	0.0375	162.887	194.774	193.501

**Table 12:** ARLs of a balanced case for *l* = 4.

Shift		Kernel		
*δ*_*S*_	*δ*_*μ*_	RBF	Polynomial	Linear
0	0	**373.580**	**380.340**	**372.780**
0.1	0.0025	355.515	439.030	414.945
0.2	0.0050	345.457	432.473	404.050
0.3	0.0075	322.988	421.480	398.750
0.4	0.0100	317.214	410.244	389.146
0.5	0.0125	306.588	387.608	373.783
0.6	0.0150	287.846	366.947	351.531
0.7	0.0175	276.688	349.161	332.004
0.8	0.0200	260.716	329.830	311.562
0.9	0.0225	249.611	311.397	294.925
1.0	0.0250	239.357	296.359	278.536
1.1	0.0275	228.161	281.018	262.665
1.2	0.0300	218.475	265.932	248.548
1.3	0.0325	208.885	252.933	235.671
1.4	0.0350	200.093	241.224	224.245
1.5	0.0375	191.384	230.123	212.913

#### 5.2.4 Comparison with PCA Mix chart

**Figure 3:**
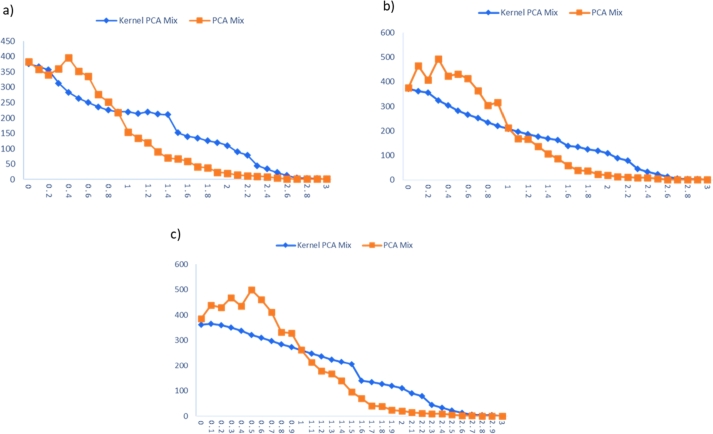
ARLs comparison for extreme imbalanced case for: a) *p* = 5, *l* = 2, b) *p* = 5, *l* = 3, and c) *p* = 5, *l* = 4.

**Figure 4:**
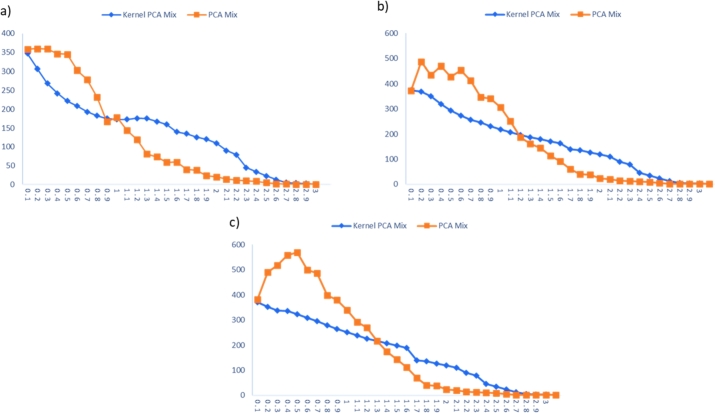
ARLs comparison for imbalanced case: a) *p* = 5, *l* = 2, b) *p* = 5, *l* = 3, and c) *p* = 5, *l* = 4.

**Figure 5:**
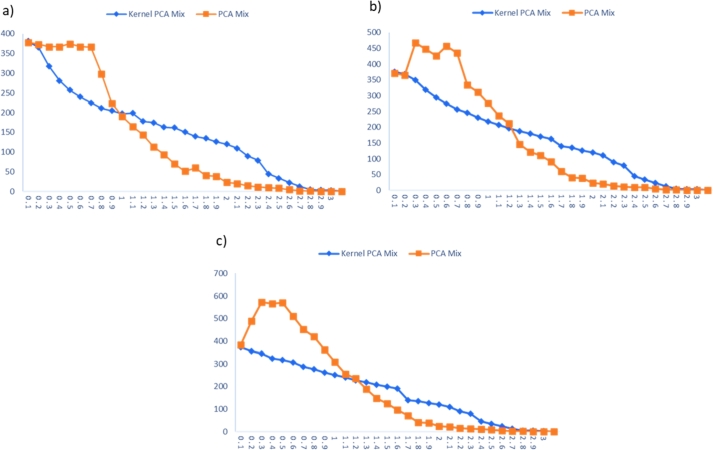
ARLs comparison for balanced case: a) *p* = 5, *l* = 2, b) *p* = 5, *l* = 3, and c) *p* = 5, *l* = 4.

The Kernel PCA Mix performance chart is compared with the performance of the PCA Mix chart in inspecting the nonlinear data. The performance comparisons for extreme imbalanced, imbalanced, and balanced cases are tabulated in Table 13, Table 14, Table 15, respectively. Meanwhile, the visualizations of these comparisons are displayed in Figure 3, Figure 4, Figure 5.

**Table 13:** Performance comparison between KPCA Mix and PCA Mix charts for extreme imbalanced case.

Shift		p=5, l=2		p=5, l=3		p=5, l=4	
*δ*_*S*_	*δ*_*μ*_	KPCA Mix	PCA Mix	KPCA Mix	PCA Mix	KPCA Mix	PCA Mix
0	0	**376.820**	**383.490**	**370.920**	**376.110**	**362.860**	**385.690**
0.1	0.0025	367.375	358.360	361.410	465.410	365.750	438.810
0.2	0.0050	357.063	340.610	356.220	408.150	359.500	430.130
0.3	0.0075	313.003	361.040	323.920	493.960	350.493	469.200
0.4	0.0100	284.322	397.270	303.690	424.150	338.492	436.240
0.5	0.0125	264.272	352.370	281.498	430.750	321.630	499.830
0.6	0.0150	250.244	335.160	267.280	413.010	311.320	461.580
0.7	0.0175	236.421	276.230	252.489	364.630	297.746	411.360
0.8	0.0200	226.051	253.160	235.111	303.430	285.544	332.780
0.9	0.0225	220.402	217.230	220.115	315.980	274.721	328.360
1.0	0.0250	219.707	154.640	207.927	213.670	260.177	263.660
1.1	0.0275	224.183	134.610	196.856	169.880	248.052	212.700
1.2	0.0300	239.949	120.240	186.622	166.900	236.200	177.520
1.3	0.0325	272.919	89.690	177.566	136.860	224.626	166.600
1.4	0.0350	210.705	70.400	169.523	107.190	214.449	140.340
1.5	0.0375	152.232	67.120	162.292	87.070	205.602	95.630

**Table 14:** Performance comparison between KPCA Mix and PCA Mix charts for imbalanced case.

Shift		p=5, l=2		p=5, l=3		p=5, l=4	
*δ*_*S*_	*δ*_*μ*_	KPCA Mix	PCA Mix	KPCA Mix	PCA Mix	KPCA Mix	PCA Mix
0	0	**386.060**	**360.580**	**374.770**	**372.990**	**371.100**	**381.750**
0.1	0.0025	346.665	358.310	368.130	487.140	351.615	490.200
0.2	0.0050	306.600	359.580	349.987	435.500	337.440	518.210
0.3	0.0075	268.633	359.080	318.833	470.580	335.985	557.740
0.4	0.0100	242.388	346.050	294.130	427.430	322.286	569.470
0.5	0.0125	222.198	345.080	274.145	452.800	308.940	500.090
0.6	0.0150	208.613	302.500	256.280	412.790	296.383	487.080
0.7	0.0175	193.365	279.090	245.261	346.090	279.708	398.220
0.8	0.0200	182.924	231.490	230.261	340.540	264.274	379.700
0.9	0.0225	175.184	166.520	218.263	306.790	251.411	339.520
1.0	0.0250	172.916	178.650	207.781	250.840	238.127	292.030
1.1	0.0275	172.819	143.750	196.715	186.980	226.427	268.970
1.2	0.0300	176.240	119.500	187.601	162.270	217.344	216.290
1.3	0.0325	175.111	81.310	178.948	145.640	207.195	174.670
1.4	0.0350	167.725	73.920	170.626	112.920	197.691	143.190
1.5	0.0375	159.685	58.780	162.887	91.410	188.935	112.000

**Table 15:** Performance comparison between KPCA Mix and PCA Mix charts for balanced case.

Shift		p=5, l=2		p=5, l=3		p=5, l=4	
*δ*_*S*_	*δ*_*μ*_	KPCA Mix	PCA Mix	KPCA Mix	PCA Mix	KPCA Mix	PCA Mix
0	0	**380.770**	**378.110**	**374.770**	**370.220**	**373.580**	**383.910**
0.1	0.0025	364.740	373.140	368.130	365.360	355.515	488.570
0.2	0.0050	317.727	366.600	349.987	466.790	345.457	572.220
0.3	0.0075	281.193	366.600	318.833	447.910	322.988	565.340
0.4	0.0100	257.002	374.300	294.130	425.940	317.214	570.590
0.5	0.0125	239.968	367.060	274.145	456.440	306.588	509.660
0.6	0.0150	224.706	366.260	256.280	434.600	287.846	451.400
0.7	0.0175	210.456	298.540	245.261	334.870	276.688	419.120
0.8	0.0200	204.304	223.350	230.261	310.620	260.716	362.910
0.9	0.0225	197.367	189.670	218.263	276.670	249.611	307.540
1.0	0.0250	198.296	164.760	207.781	236.940	239.357	255.030
1.1	0.0275	177.847	143.490	196.715	212.420	228.161	235.770
1.2	0.0300	174.638	113.170	187.601	145.390	218.475	187.540
1.3	0.0325	163.244	94.000	178.948	121.600	208.885	147.950
1.4	0.0350	161.971	69.930	170.626	110.920	200.093	123.910
1.5	0.0375	150.653	51.270	162.887	90.500	191.384	95.890

### 5.3 Discussion

In this subsection, some discussion about the performance of the proposed chart is provided. First, the best kernel used is the RBF kernel. This happened because the other kernel is developed based on a linear kernel. As we know that the process is generated to follow the nonlinear relationship. The RBF kernel is renowned to have a better performance in inspecting the nonlinear process and under general smoothness assumptions (Zhicheng et al., 2012). Therefore, it makes sense that the RBF kernel performs better in this study.

Table 16 tabulates the summary of the performance comparison between the Kernel PCA Mix chart and PCA Mix chart. In general, both charts yield good performance in detecting the process shift. However, for the specific case, the Kernel PCA Mix chart demonstrates better performance for the small process shift. Meanwhile, the PCA Mix chart has a better performance for a large shift in process. This result indicates that the proposed method is better to be used for nonlinear data with a small shift. This happened because the PCA Mix chart is only developed for the linear process. In contrast, the proposed Kernel PCA Mix chart is developed to overcome the nonlinearity problem so that it has good performance.

**Table 16:** Summary of performance comparison.

Parameter data non-metric	*l*	Kernel PCA Mix	PCA Mix
*θ*_1_,*θ*_2_ = 0.3 and *θ*_3_ = 0.4	2	•	
	3	•	
	4	•	

*θ*_1_,*θ*_2_ = 0.1 and *θ*_3_ = 0.8	2	•	
	3	•	
	4	•	

*θ*_1_,*θ*_2_ = 0.05 and *θ*_3_ = 0.9	2	•	
	3	•	
	4	•	

• represents better performance for a small shift.

represents better performance for a large shift.

## 6 Application to the real data

**Figure 6:**
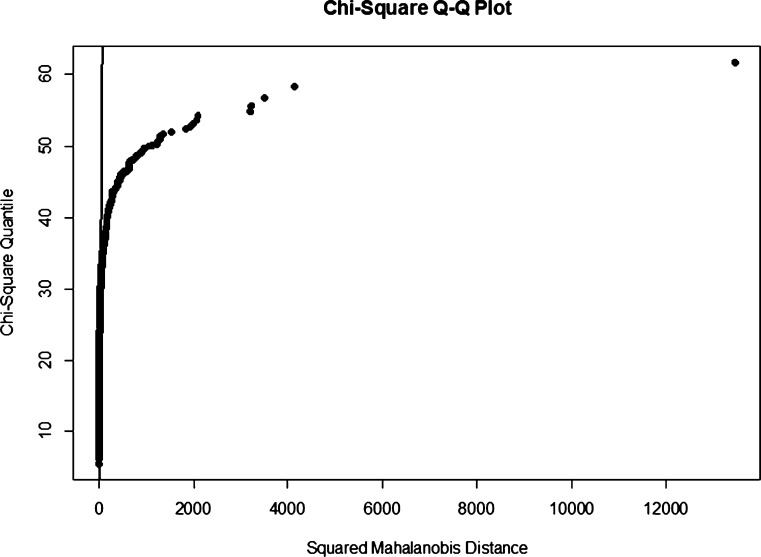
NSL-KDD 20% QQ Plot for normal connection.

In this section, the Kernel PCA Mix chart is applied to monitor intrusion in the real dataset. The dataset used is the famous NSL KDD. This research only analyzes 20% of the NSL KDD dataset which can be found at https://www.unb.ca/cic/datasets/nsl.html. The summary of this dataset is displayed in Table 17. From Fig. 6, it is known that the normal connection of the NSL KDD dataset is not normally distributed. The RBF kernel is used in this analysis due to its performance consistency in simulation studies.

**Table 17:** Summary of NSLKDD 20% dataset.

Attack types	Number of observations	Percentage (%)
Normal	13,449	53.39

DOS	9,234	36.65
Probe	2,289	9.09
U2R	11	0.04
R2L	209	0,83

**Total**	25,192	100.00

Table 18 shows the accuracy rate of the Kernel PCA Mix chart in detecting intrusion in the NSL KDD dataset for several principal component scores. From the results, it is seen that the optimal number of principal components is 4. After finding the optimal number of principal components, this analysis is continued by searching for the optimal value of *σ*. Based on the result in Table 19, it can be known that the optimal value of *σ* is 0.001. From the detection results, it can be seen that the proposed method has a detection accuracy of about 0.85769. The misdetection happens due to the large value of the FN rate which indicates that more attacks cannot be accurately detected as the real attack.

**Table 18:** Performance of Kernel PCA Mix Control Chart in monitoring the NSL-KDD dataset for different numbers of principal components.

*l*	Accuracy	FP rate	FN rate
2	0.82744	0.06751	0.29285
3	0.84741	0.06714	0.25044
4	**0.85769**	0.08305	0.21016
5	0.84653	0.07361	0.24491
7	0.82347	0.13183	0.22771
10	0.84741	0.06714	0.25044
20	0.68986	0.42724	0.17601

**Table 19:** Performance of Kernel PCA Mix Control Chart in monitoring the NSL-KDD dataset for *l* = 4 and several values of *σ*.

*σ*	Accuracy	FP rate	FN rate
0.10000	0.58772	0.02632	0.85429
0.01000	0.84522	0.06825	0.25385
0.00100	**0.85769**	0.08305	0.21016
0.00500	0.84590	0.06022	0.26160
0.00010	0.63492	0.52643	0.18027
0.00001	0.53385	0.00000	1.00000

The performance comparison with the other methods is shown in Table 20. The proposed method is compared with several machine learning algorithms (Decision Tree, Naïve Bayes, Logistic Regression, and Support Vector Machine) and control chart method (Hotelling's $T^{2}$ and PCA Mix chart). According to the table, it is clear that the proposed method has higher accuracy compared to the other machine learning methods and control chart method for the same number of quality characteristics monitored. Also, we can see that the proposed method yields a lower FP rate. This is indicating that the proposed method produces a lower false alarm.

**Table 20:** Performance comparison with the other methods.

Method	Accuracy	FP rate
Hybrid Decision Tree (Farid et al., 2014)	0.8192	0.1740
Hybrid Naïve Bayes (Farid et al., 2014)	0.8239	0.1640
Logistic Regression (Belavagi and Muniyal, 2016)	0.8400	0.1700
Support Vector Machine (Belavagi and Muniyal, 2016)	0.7500	0.2400
Hotelling's *T*^2^ chart	0.7023	0.1433
PCA Mix	0.8041	0.3171
**Proposed method**	**0.8577**	**0.0831**

## 7 Conclusion and future research

In this research, the control chart which has the ability in monitoring the mixed variable and attribute characteristics with nonlinear relationships is proposed. The performance of the proposed chart is evaluated for several types of attribute characteristics and several kernel functions. Through simulation studies, it can be seen that the Kernel PCA Mix chart can detect the shift in process. It also can be known that the better kernel function is RBF due to its consistency in detecting a shift in process. The comparison with the PCA Mix chart shows that the proposed chart has better performance for a small shift in the process. On the other hand, the PCA Mix chart has better performance for a large shift. This method can be applied in monitoring the process with a nonlinear relationship such as in manufacture and industry, chemical process, biological process, and network anomaly detection. Furthermore, the proposed chart is also applied to monitor the real dataset. The well-known NSL KDD dataset is used as the benchmark for the proposed chart. The monitoring results show that the proposed chart has a good accuracy detection at about 0.85769. Compared to the other methods the proposed demonstrates a better performance by producing higher accuracy and lower false alarms. For future research, the Generative Principal Component Analysis (K. Liu et al., 2020, 2021) can be used in order to improve the performance of the proposed method. Also, the Bayesian-based PCA method (Y. Liu et al., 2018) can be applied for imbalanced cases.

## Declarations

### Author contribution statement

Muhammad Ahsan: Conceived and designed the experiments; Performed the experiments; Analyzed and interpreted the data; Wrote the paper.

Muhammad Mashuri: Conceived and designed the experiments; Wrote the paper.

Hidayatul Khusna: Analyzed and interpreted the data; Wrote the paper.

Wibawati: Analyzed and interpreted the data; Contributed reagents, materials, analysis tools or data.

### Funding statement

This work was supported by 10.13039/501100005981Direktorat Jenderal Pendidikan Tinggi (3/81/KP.PTNBH/2021).

### Data availability statement

Data associated with this study is available at https://www.unb.ca/cic/datasets/nsl.html.

### Declaration of interests statement

The authors declare no conflict of interest.

### Additional information

There is no additional information.

## Appendix A Source code

rm(list=ls(all=TRUE)) alpha=0.00273 uclt=15942.24 n=1000 n1=70 n2=30 p=5 k=2 q=3 rho=0.3 p0=0.3 sigmaz=0.001 degreez=1 UCL0=NULL t2pc=NULL t2pctr=NULL kdhsl=NULL ucl1=NULL shift=0.0025 t2pctr=NULL RL=NULL daq=NULL eig=NULL mup=NULL for (ju in 1:2){ t2pc=NULL t2pctr=NULL Sigma <- matrix(rho,p,p)-diag(rho,p)+diag(1,p) mu<-rep(0, p) a1=mvrnorm(n,mu,Sigma) prob=c(rep(p0,q-1),1-sum(rep(p0,q-1))) rcat=t(rmultinom(n, 1,prob)) a=data.frame(cbind(a1, rcat)) kpca<-kpca(~.,data=a,kernel="rbfdot", kpar=list(sigma=sigmaz),features=k) #kpca<-kpca(~.,data=a,kernel="polydot", kpar=list(degree=degreez, scale=1, offset=0), features=k) #kpca<-kpca(~., data=data.frame(a), kernel="tanhdot", kpar=list(scale=1, offset=1), features=k) #pcs=pcv(kpca) pcs=rotated(kpca) eig1=as.vector(kpca@eig) eig=rbind(eig,eig1) siglam=diag(eig1,k,k) mup1=colMeans(pcs) mup=rbind(mup,mup1) for(ka in 1:n) { t2pcs=pcs[ka,]%*%solve(siglam)%*%(transpose (pcs[ka,])) t2pctr=rbind(t2pctr,t2pcs) } #plot(t2pctr) #hist(t2pctr) } eigen=mean(eig) mup2=mean(mup) Sigma <- matrix(rho,p,p)-diag(rho,p)+diag(1,p) mu<-rep(0, p) mu1<-rep(2,p) a1=mvrnorm(n1,mu,Sigma) a2=mvrnorm(n2,mu1,Sigma) prob=c(rep(p0,q-1),1-sum(rep(p0,q-1))) rcat1=t(rmultinom(n1, 1,prob)) rcat2=t(rmultinom(n2, 1,prob)) aa=data.frame(cbind(a1, rcat1)) ab=data.frame(cbind(a2, rcat2)) a0=rbind(aa,ab) pcs=predict(kpca,a0) eig1=as.vector(kpca@eig) mup1=colMeans(rotated(kpca)) mup=rbind(mup,mup1) eig=rbind(eig,eig1) siglam=diag(eig1,k,k) t2pc=NULL t2pctr=NULL for(ka in 1:(n1+n2)) { t2pcs=pcs[ka,]%*%solve(siglam)%*%(transpose (pcs[ka,])) t2pctr=rbind(t2pctr,t2pcs) } plot(t2pctr) lines(t2pctr) lines(rep(uclt,n1+n2), col="blue")
